# The Suppression of WRKY44 by GIGANTEA-miR172 Pathway Is Involved in Drought Response of *Arabidopsis thaliana*

**DOI:** 10.1371/journal.pone.0073541

**Published:** 2013-11-06

**Authors:** Yingying Han, Xuan Zhang, Yaofeng Wang, Feng Ming

**Affiliations:** 1 State Key Laboratory of Genetic Engineering, Institute of Genetics, Fudan University, Shanghai, China; 2 Institute of Plant Biology, School of Life Science, Fudan University, Shanghai, China; Ghent University, Belgium

## Abstract

Water availability is an important environmental factor that controls flowering time. Many plants accelerate flowering under drought conditions, a phenomenon called drought escape. Four pathways are involved in controlling flowering time, but which ones participate in drought escape is not yet known. In this study, plants with loss-of-function mutations of *GIGANTEA (GI*) and *CONSTANS* (CO) exhibited abnormal drought-escape phenotypes. The peak mRNA levels of *GI* and FKF1 (Flavin-binding Kelch domain F box protein 1) and the mRNA levels of *CO* and FT (Flowering locus T) changed under drought stress. The microRNA factor *miRNA172E* was up-regulated by drought stress, and its up-regulation was dependent on *GI*, while other *miRNA172s* were not. Water-loss analyses indicated that *gi* mutants were more sensitive while *miRNA172* over-expressing (*miRNA*172-OX) plants were less so to drought stress than wild-type plants. Digital gene expression and real-time PCR analyses showed that *WRKY44* was down-regulated by *GI* and *miRNA172*. The WRKY44 protein could interact with TOE1 (a target of *miRNA*172) in a yeast two-hybrid system. We proposed that *GI–miRNA172–WRKY44* may regulate drought escape and drought tolerance by affecting sugar signaling in *Arabidopsis*.

## Introduction

Unlike most animals, plants are sessile organisms. They cannot move to escape the biotic and abiotic stresses that threaten them throughout their life cycles. To adapt to unfavorable and sometimes unexpected conditions, plants have evolved many flexible survival strategies, one of which is the control of flowering time [1,2]. Flowering time is finely tuned because it is of critical importance to successful reproduction and maximal seed set [3].

Flowering time is regulated by multiple environmental and endogenous factors [4]. In general, these factors can be grouped into four genetic pathways: the photoperiod, phytohormone, vernalization, and autonomous pathways [5,6]. These pathways ultimately crosstalk at common targets, such as *Flowering Locus T* (*FT*) and *Leafy*, to promote the transition from vegetative to reproductive phase [4,7]. Also, several microRNAs (miRNAs) participate in these pathways to maintain homeostasis and accurate flowering time, i.e., miRNA159 in the phytohormone pathway [8], miRNA156 in the autonomous pathway [9,10], and miRNA172 in the photoperiod pathway. Notably, miRNA156 inhibits the transcription of miRNA172b via SPL9 and, redundantly, SPL10 [11]. 

Beside day length [2], phytohormones [12], and vernalization [13], other environmental pressures affect flowering time, including sub-optimal temperature, light quality, oxidative stress, and osmotic stress, via known genetic factors [14-16]. For example, Blazquez proposed that a thermosensory pathway controls flowering time, in which suboptimal temperatures (i.e., 16°C; the optimumal temperature is 23°C ) can inhibit flowering. He proved that ambient temperature affected flowering dependent on FLC (Flowering Locus C) [17]. Strasser proved that the photoperiod pathway, independently mediated by *ELF3* and *TFL1* affecting expression of *SOC1*, also participated in the thermosensory pathway [15].

In this study, we considered the regulation of flowering time under drought stress. As the greenhouse effect causes global climate warming, drought is becoming a major agronomic threat to crop yields [18,19]. Under excessively dry conditions, plants must balance drought resistance and escape (via reproduction) to maximize the probability of genetic survival. Thus, water availability affects flowering time in many angiosperms [14]. Many terrestrial plants flower earlier when water is deficient, a phenomenon well studied in wheat, *Brassica*, and *Arabidopsis* [20,21], but which pathway is involved in drought escape is not yet clear.

Drought has been reported to alter physiological sugar levels. An increase in soluble sugar with a decrease in leaf osmotic potential was observed during drought [22]. The reduced osmotic potential may prevent moisture loss. Also, sucrose promotes flowering in many plant species [23-25]. Feeding sucrose in photosynthetic amounts reversed the floret abortion induced by drought stress [26].

Accelerated flowering under drought will reduce crop yields. Therefore our study focused on the genetic mechanism of accelerated flowering under drought stress in *Arabidopsis*. We observed and characterized drought escape and defense in different genotypes of *Arabidopsis*. Our results confirmed that photoperiod factor GIGANTEA (GI) was involved in both drought escape and drought resistance. Also, *GI–miR172* may function in sugar signaling by down-regulating *WRKY44*. This study provides a foundation for researching reduced crop yields under long-term drought.

## Materials and Methods

### Plant Materials

 The *Arabidopsis thaliana* ecotypes *Col-0* and *Ler-0* were used as wild types (WT). Mutants gi, co*, gai* were in the *Ler-0* background, and *flc-3* was in the *Col-0* background. The *miRNA172s-OX* lines were in the *Col-0* background.

### Growth Conditions and Drought Treatment

WT and mutant plants were grown in a climate-controlled culture room at 23–25°C with a relative humidity of 40–60% under long day (LD) conditions (16 h light/8 h dark). The plants were grown on a medium containing 9:3:1 vermiculite: sphagnum peatmoss: perlite. The medium was saturated with tap water containing diluted (1000-fold) Hyponex during the first watering. Thereafter, the plants were irrigated with tap water. For a control (CK), plants were thoroughly watered every 4 d without water-logging the soil. For the drought (DR) treatment, the plants were not watered until samples were collected. 

### Flowering Time Estimation

WT plants (*Col-0* and *Ler-0*) and four loss-of-function mutants were used to estimate the flowering time under drought stress. Mutants of the photoperiod pathway factor *GIGANTEA* (*gi*, CS181), the phytohormone pathway factor *Gibberellic Acid Insensitive* (*gai*, CS63), and the autonomous pathway factor *Flowering Locus C* (*flc-3*, SALK_140021) were purchased from the *Arabidopsis* Biological Resource Center (http://abrc.osu.edu/). Mutants of the photoperiod pathway factor *CO* (*CONSTANS*) was kindly provided by Hongquan Yang (Institute of Plant Physiology and Ecology, Shanghai Institutes for Biological Sciences, Chinese Academy of Sciences).

Drought treatment began about 10 d before normal (CK) flowering. Specifically, at an age of 10 d for WT, 23 d for *gi* and *co*, 10 d for *flc*, and 14 d for *gai*. The flowering time was counted in days. Three biological replicates were performed. WT (*Col-0*) was also grown under short day (SD: 8/16 h; 23–25°C, 40–60% humidity) conditions and DR treatment beginning at 35 d of age.

### Expression Analyses of Photoperoid Pathway Genes

#### Real-time PCR analysis of rhythmic expression of photoperiod pathway-related genes

Rhythmic expression of four photoperiod pathway genes, *GI*, FKF1 (Flavin-binding Kelch domain F box protein 1), *CO* (*CONSTANS*), and *FT*, was detected by real-time reverse transcription PCR (qRT-PCR). Expression of *ACTIN11* was the control for all the qRT-PCR. Primer sequences are listed in Table 1.

**Table 1:** Primers of photoperiod pathway genes for real time PCR.

Gene	AGIs(*Arabidopsis* Genome Initiative)	Forward	Reverse
*GI*	AT1G22770	GGTCGACGGTTTATCCAA TCTA	CGGACTATTCATTCCGTTCTTC[64]
*CO*	AT5G15840	CAGGGACTCACTACAACGACAATG	TCCGGCACAACACCAGTTT[65]
*FT*	AT1G65480	AGATTGGTGGAGAAG ACC	CCAGTTGTAGCAGGGATA
*FKF*	AT1G68050	GAAGTCTTCACTGGCTATCG	GATCAACCAATGGGTGACG
*ACTIN11*	AT3G12110	GTTCTTTCCCTCTACGCT	CTTACGATTTCACGCTCT

Note: *ACTIN11* was used as the control.

WT (*Ler-0*) plants were grown under LD. CK and DR treatments were performed as described above. Drought treatment began at 10 d of age and continued for 10 d. Leaf samples were collected at 4-h intervals for 72 h. Then the DR plants were recovered by watering for 5 d. Leaf samples were again collected every 4 h for 24 h. From each sample, total RNA was isolated and treated with RNase-free DNase (Promega, Beijing, China) according to the manufacturer’s recommendations. Then, 2 µg RNA was used in a reverse transcription reaction (M-MLV RTase cDNA Synthesis Kit; Takara, Kyoto, Japan) with an oligo(T) primer. For qRT-PCR, 1.5 µL of diluted cDNA (1:10) was used as template in 20-µL PCR mixtures according to the manufacturer’s instructions (SYBR Premix Ex Taq^TM^, Takara) in a 384-well quantitative PCR thermocycler (7900-HT; Applied Biosystems, Foster City, CA, USA). Cycle parameters were: 2 min at 95°C and 40× (15 s at 95°C, 15 s at 55°C; 20 s at 72°C). Three biological replicates were performed.

#### Expression of miRNA172s

*Ler-0* plants were DR treated beginning at 14 d of age. For semi-quantitative RT-PCR of primary miRNA *pri-miRNA172*, leaf samples were harvested 4 h after dawn at 2 d intervals for 8 d. There were three biological replicates. RNA preparation and cDNA analysis were carried out as described above using the primers listed in Table 2. The cycle conditions were: 3 min at 94°C; 28× (40 s at 94°C; 40 s at 55°C; 40 s at 72°C); and 5 min at 72°C. 

**Table 2:** Primers for semi-quantitative RT-PCR of *pri-miRNA172*s .

**Gene**	**Froward**	**Reverse**
***miRNA172A***	TCTGTTTTTGCTTCCCCT	TGGGATTGGCAACATAAG
***miRNA172B***	TTCACGGTCTAAAATCAGAA	TCAAGTCAAGATCAAAGGC
***miRNA172C***	AACGATTTATACAGTCTTTTG	AATCCTAAAATAATGGATCAG
***miRNA172D***	GCAAGCTTTAATGCTTGTGGGCTACG	CAACAGACATATACATGCTCC
***miRNA172E***	CCTTTGGCTTCTGTTCCTGAC	TCTTCCTCGGTCAATGAAACTAT
***ACTIN 11***	TGGTTGGTATGGGACAAAAAG	AGGTAATCAGTAAGGTCACGG

To assay mature miRNAs, treatments were as for semi-quantitative analysis of *pri-miRNA172*, and samples were collected after 8 d of DR treatment. Both Ler-0 and the *gi* mutant were examined. Each treatment was replicated twice, with three samples per replicate. RNA was prepared as described above. cDNA was synthesized using gene specific primers: U6 5’ATT TGG ACC ATT TCT CGA TTT GT3’; miRNA172A/B 5’ATT TGG ACC ATT TCT CGA TTT GT 3’; miRNA172C/D 5’GTC GTA TCC AGT GCG TGT CGT GGA GTC GGC AAT TGC ACT GGA TAC GAC CTG CAG 3’; and miRNA172E 5’GTC GTA TCC AGT GCG TGT CGT GGA GTC GGC AAT TGC ACT GGA TAC GAC ATG CAG3’. The SYBR method (2X SuperArray PCR master mix (Cat. No. PA-112, SABiosciences, Valencia, CA, USA) was used in an ABI PRISM 7900 system (Applied Biosystems). U6 was the internal control. Primers for the following PCRs are shown in Table 3. Reaction conditions were as follows: 10 min at 95°C and 40× (15 s at 95°C; 60 s at 60°C); the annealing temperature for *miRNA172* was 60°C. For the melting curve, the reaction conditions were as follows: 2 min at 95°C; 20 s at 60°C; 10 s at 99°C, decreasing to 60°C.

**Table 3:** Primers for microRNA assay of mature *miRNA172*s.

miRNA	sequences	Product length (bp)
U6	F:5’CGATAAAATTGGAACGATACAGA3’R:5’ATTTGGACCATTTCTCGATTTGT3’	82
ath-*miRNA172A/B*	GSP:5’GGGGAGAATCTTGATGATG3’ R:5'CAGTGCGTGTCGTGGAGT3'	65
ath- *miRNA 172C/D*	GSP:5’GGGGAGAATCTTGATGATG3’ R:5'CAGTGCGTGTCGTGGAGT3'	65
ath- *miRNA 172E*	GSP:5’GGGGGAATCTTGATGATG3’ R:5'CAGTGCGTGTCGTGGAGT3'	64

Note: GSP, Gene specific primer; R: Reverse primer.

### Measurement of Transpiration Rate and Water Loss

*Arabidopsis* lines (*Col-0* ecotype) over-expressing *miRNA172* were used in this study. The primers for gene amplification and enzymes for cloning are listed in Table 4. The *miRNA172* fragments were cloned into pCAMBIA1301 expression vector and transgenesis was carried out by the floral-dip method mediated by *Agrobacterium* [27]. Seeds of transgenic lines over-expressing *miRNA172* (*miRNA172*-OX) were selected on MS agar medium with 20 mg/L hygromycin. E1-2 (*miRNA172e* over-expressing), D6-3 (*miRNA172d* over-expressing), and A1-10 (*miRNA172a* over-expressing) were transgenic homozygote lines. 

**Table 4:** Primers of miRNA172 amplification for transgenic plants.

Forward	5’ → 3’
*miRNA172D*-F(*Hind* III)	GCAAGCTTTAATGCTTGTGGGCTACG
*miRNA172D*-R(*BamH* I)	GCGGATCCCAACAGACATATACATGCTCC
*miRNA172E*-F(*Hind* III)	GCAAGCTTCCTTTGGCTTCTGTTCCTGAC
*miRNA172E*-R(*Sac* I)	GCGAGCTCTCTTCCTCGGTCAATGAAACTAT
*miRNA172A*-F(*BamH* I)	GCGGATCCTCTGTTTTTGCTTCCCCT
*miRNA172A*-R(*Pst* I)	GCCTGCAGTGGGATTGGCAACAT AAG

*Ler-*0, *gi*, A1-10 (*miRNA172*a-OX), D6-3 (*miRNA172d*-OX), and E1-2 (*miRNA172e* -OX) were either CK or DR treated at 10 d of age. Samples were collected after 10 d. Leaves of similar developmental stage (3^rd^–5^th^ true rosette leaves) were collected and placed abaxial-side up on open Petri dishes. Transpiration rate and water loss were measured according to Kang et al. [28]. Briefly, the leaves were weighed at hourly intervals. The transpiration rate was represented by the change in weight over time for CK-treated plants, i.e., weight/(fresh weight), while water loss was represented by the lost weight for the DR-treated plants, i.e., (fresh weight – weight)/(fresh weight). Three biological replicates were performed. 

### Digital Gene Expression Analysis of *gi* under Drought

WT (*Ler*-0) plants and *gi* mutants were CK or DR treated as described above for the qRT-PCR analyses. Samples were collected from two independent treatments. Then digital gene expression (DGE) analysis was performed with all four combinations of genotype and treatment. In detail, we extracted 6 μg of total RNA, purified mRNA via Oligo(dT) magnetic bead adsorption, then used Oligo(dT) to guide reverse transcription to synthesize double-stranded cDNA. NlaIII was used to cut the CATG sites in the cDNA, then cDNA fragments with 3' ends were purified with magnetic-bead precipitation, and Illumina adapter 1 (Illumina, San Diego, CA, USA) was added to their 5' ends. The junction of Illumina adapter 1 and the CATG site is the recognition site of *MmeI*, which cuts 17 bp downstream of the CATG site, producing tags with adapter 1. After removing 3' fragments via magnetic-bead precipitation, Illumina adapter 2 was introduced at the 3' ends of tags, producing tags with different adapters at their ends to form a tag library. After 15 cycles of linear PCR amplification, 85 base strips were purified by 6% TBE polyacrylamide gel electrophoresis. These strips were then digested, and the single-chain molecules were fixed onto the Solexa Sequencing Chip (flowcell). Each molecule grew into a single-molecule cluster sequencing template through *in situ* amplification. Then, labeled nucleotides were added and sequencing by synthesis was performed. Each tunnel generated millions of raw reads 35 bp length. The raw data were normalized by the number of tags per million. The main reagents and supplies were Illumina Gene Expression Sample Prep Kit and Solexa Sequencing Chip (flowcell), and the main instruments were Illumina Cluster Station and Illumina Genome Analyzer System. 

We focused on genes that showed log_2_ ≥ 1 (the relative expression levels between WT and *gi* under DR) with false discovery rate values ≤ 0.001. 

### Expression Analysis of *WRKY*s

The expressions of *WRKY* family members including *WRKY*19, 20, 40, 44, 51, 54, 65, 72, and 74 were examined by qRT-PCR as described above. The primers are listed in Table 5.

**Table 5:** Primers of *WRKY* genes for real time PCR.

Gene	AGIs	Forward	Reverse
WRKY19	AT4G12020	CGATTTATGCCTCCGAAG	CGACTTGTTGTATCCATTC
WRKY20	AT4G26640	CGCCGAAACTCTGGTGGTATG	TGACGCTGCCGCTTCTCC
WRKY40	AT1G80840	TCACTATTGGCGTTACTCGTATG	CCTCTCGGTTATGTTGCTCTTG
WRKY74	AT5G28650	AACAAGATTGCGGACATACC	GCC TTC ATA AGT CAC AAT AAGC
WRKY72	AT5G15130	TGT GTT AGA GCA AGA TGT G	CAT AGG TTG TGA TTA GTA TAG AC
WRKY65	AT1G29280	ACCAAATTCTTCAACCTTTAACG	TTGTGCCGAGATCCTTCC
WRKY51	AT5G64810	ATCTCATCTCCGACAAGCATC	AACCATCATCCATCACATCAATC
WRKY54	AT2G40750	CCGTCGCCGTCTCTGTCC	TCTCGTCTTTCTAGTGTAGCATCC
WRKY44	AT2G37260	CGAGATTGTAGACGCTGCTATAAG	AGAGACGGTTGCTTTGGAGAC

### Phylogenetic Analysis

We performed phylogenetic analysis on the sequences listed in Table 6. Sequences were aligned using ClustalX 1.8 [29] and a phylogenetic tree was constructed with MEGA5 [30] using neighbor joining method [31]. 

**Table 6:** *WRKY* sequences used in phylogenetic analysis.

Gene symbol	Accession number
*Arabidopsis thaliana*	
*AtWRKY19*	NM_001160750
*AtWRKY20*	NM_179119
*AtWRKY21*	NM_128611
*AtWRKY40*	NM_106732
*AtWRKY44*	NM_129282
*AtWRKY50*	NM_122518
*AtWRKY51*	NM_125877
*AtWRKY54*	NM_129637
*AtWRKY65*	NM_102668
*AtWRKY72*	NM_121517
*AtWRKY74*	NM_122748
*Hordeum vulgare*	
*HvWRKY5*	AJ853841
*HvWRKY9*	DQ840408
*HvWRKY32*	DQ863116
*HvWRKY34*	DQ863118
*HvWRKY37*	DQ863121
*HvWRKY41*	DQ863124
*HvWRKY46*	AY323206
*Oryza sativa*	
*OsWRKY27*	BK005030

### Yeast Two-hybrid System

The yeast host strain Y2H Gold (Clontech, Mountain View, CA, USA) was transformed with pGBKT7-TOE1 as the bait. The Y187 strain was transformed with the plasmid pGADT7 with a full-length open reading frame of WRKY20, WRKY44, or WRKY74; an empty pGBKT7 was the control. Transformants with BD (Binding Domain) and AD (activation domain) were mated on 2× YPDA medium at 30°C [32]. Mated colonies were picked and mixed with 5 mL 0.9% NaCl, then spotted on SD/–Leu/–Trp/–His/–Ade/X-α-gal/AbA agar media. The plates were cultured at 30°C and photographed after 2–3 d.

### ABA Treatment

 The two-weeks old seedlings were treated with ABA (50μM) or ddH2O. Samples were collected every 12hr, from 0 to 48hr. FT was analyzed by relative-quantitative RT- PCR. PCR conditions were as following: 3 min at 94°C; 28× (40 s at 94°C; 30 s at 55°C; 15s at 72°C); 5 min at 72°C. The primer for *FT* and *ACTIN11* were the same as that for the real time PCR.

### Statistical Analyses

Because we compared two treatments (CK and DR) with small sizes and equal variances, *t*-tests were used for all statistical tests of differential gene expression.

## Results

### The Photoperiod Pathway Mediated by *GIGANTEA* Might Be Involved in Early Flowering under Drought Stress

We examined the drought escape of *Arabidopsis* carrying mutations in genes of different flowering pathways. Flowering time (mean ± SE of three replicates, with three samples per replicate) was calculated as days after germination. Plants were either watered normally (CK) or deprived of water (DR) beginning about 10 d before normal (CK) flowering. Relative humidity throughout the experiment ranged from 40–60%.

**Figure 1:**
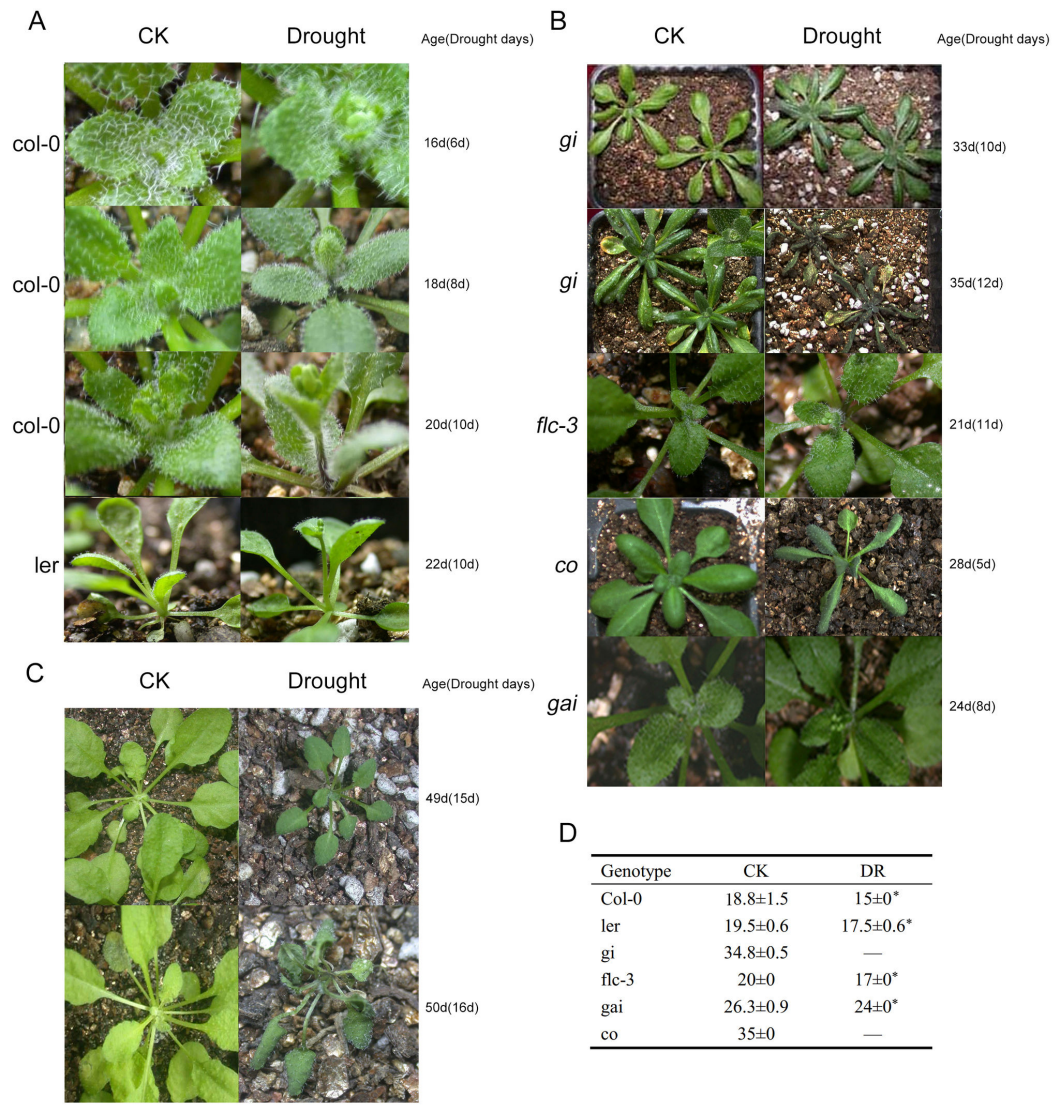
Flowering times of *Arabidopsis* wild-type (WT) and mutants of different flowering pathways under drought stress. (**A**) Early flowering of WT (Col-0 and Ler-0) plants under drought stress and long-day conditions. (**B**) Flowering times of mutants of the photoperiod (*gi*, *co*), autonomous (flc-3), and phytohormone (gai) pathways under drought stress and long-day conditions. (**C**) Flowering times of WT (Col-0) plants under drought stress and short-day conditions. (**D**) Counted flowering times (days) of plants with different genotypes under CK and DR conditions. * flowering significantly earlier under DR condition than under CK condition. DR : Drought treatment began from 10days before flowering.

In this experiment, WT *Col-0* and *Ler-0* plants under LD conditions flowered significantly earlier (*P*<0.05) under DR than under CK (Figure 1A,D). The *gai* and *flc* mutants also flowered significantly earlier (*P*<0.05) under DR, but flowering of the *gi* and *co* mutants was not induced by drought (Figure 1B,D). The *gi* and *co* plants withered after 10 d of DR. Because the onset and duration of drought treatment (lasting 10 d and beginning 10 d before normal flowering) of *gi* and *co* was the same as that of WT and other plants, these results indicated that the photoperiod pathway might be involved in early flowering under drought. WT plants did not flower earlier under DR and SD conditions (Figure 1C), indicating that day length is important for early flowering under drought.

### Expressions of Photoperiod Pathway Genes Changed under Drought Conditions

**Figure 2:**
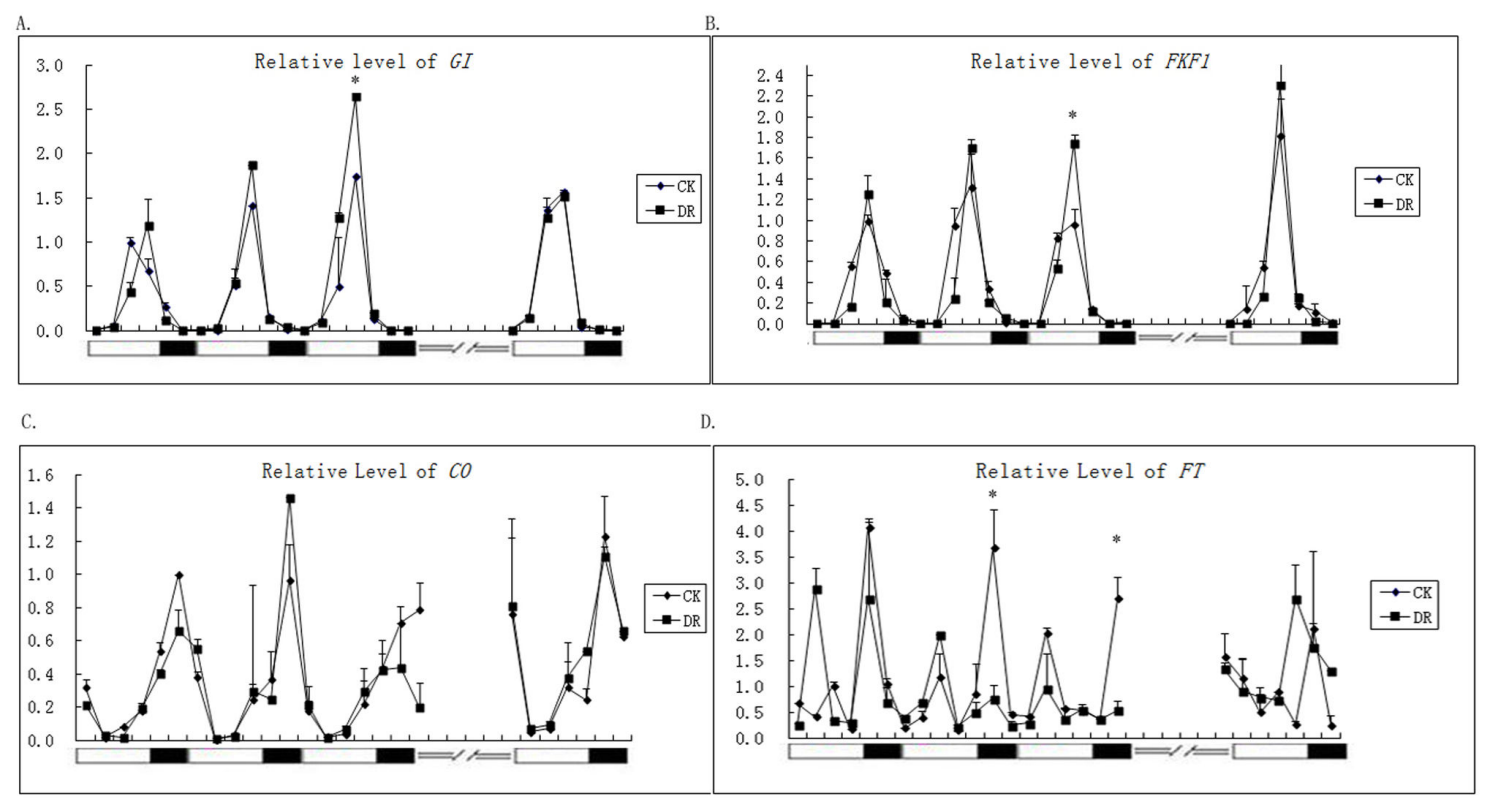
Abundance of mRNAs of flowering-time and circadian-clock–regulated genes in *Arabidopsis* under long-day control (CK) and drought (DR) conditions. The expressions of *GI* (**A**), *FKF1* (**B**), *CO* (**C**), *FT* (**D**) were analyzed by real time-PCR in Ler-0 plants grown in LDs. For each gene, the first peak on the first day under CK conditions was standardized to a level of 1. Open and closed bars along the horizontal axis represent light and dark periods, respectively, measured in hours from dawn. Each experiment was done twice with similar results. ===/ /=== represents the 5-d recovery period with watering. * indicated a significant difference (P<0.05). DR: Drought treatment began from the 10^th^ day age and maintained for 10 days.

Genes in the photoperiod pathway are transcribed rhythmically. Several important genes, including *GI*, *CO*, and *FT*, are the main factors in this pathway. A recent study indicated that *FKF1* cooperated with *GI* to activate *CO* [33]. Therefore, changes in the mRNA levels of *GI*, *CO*, *FT*, and *FKF1* were detected by qRT-PCR (Figure 2). The peak levels of *GI* and *FKF1* mRNAs were up-regulated under drought stress, while the expressions of *CO* and *FT* were reduced.

*GI* expression peaked 4 h before dusk under both CK and DR (Figure 2A). The maximum level under DR was significantly higher than that of CK. After 5 d of recovery with watering, both mRNA rhythm and levels were similar in DR plants to CK plants. *FKF1* was also up-regulated under DR (Figure 2B). Unlike *GI*, *FKF1* expression was developmentally controlled, because it was up-regulated with developmental age under both CK and DR. After recovery for 5 d, the level of *FKF1* under DR was substantially higher than that under CK, which may indicate that DR accelerated aging. 

For *CO* under CK, there was one peak within a 24-h cycle (Figure 2C). The rhythm was not changed under DR during the first 2 d of sample collection (days 11 and 12 of DR), but on the 13^th^ day of DR treatment, the expression at the peak (late) time was reduced. After recovery, the circadian expression of *CO* was recovered. *FT* was apparently down-regulated with the intensified DR condition (Figure 2D). There were two expression peaks for *FT* during one 24-h cycle. These two peaks were reduced and ultimately disappeared under DR. When water was restored, the transcription of *FT* was recovered.

### *GI* Promoted the Level of *miRNA172E* under Drought Conditions

**Figure 3:**
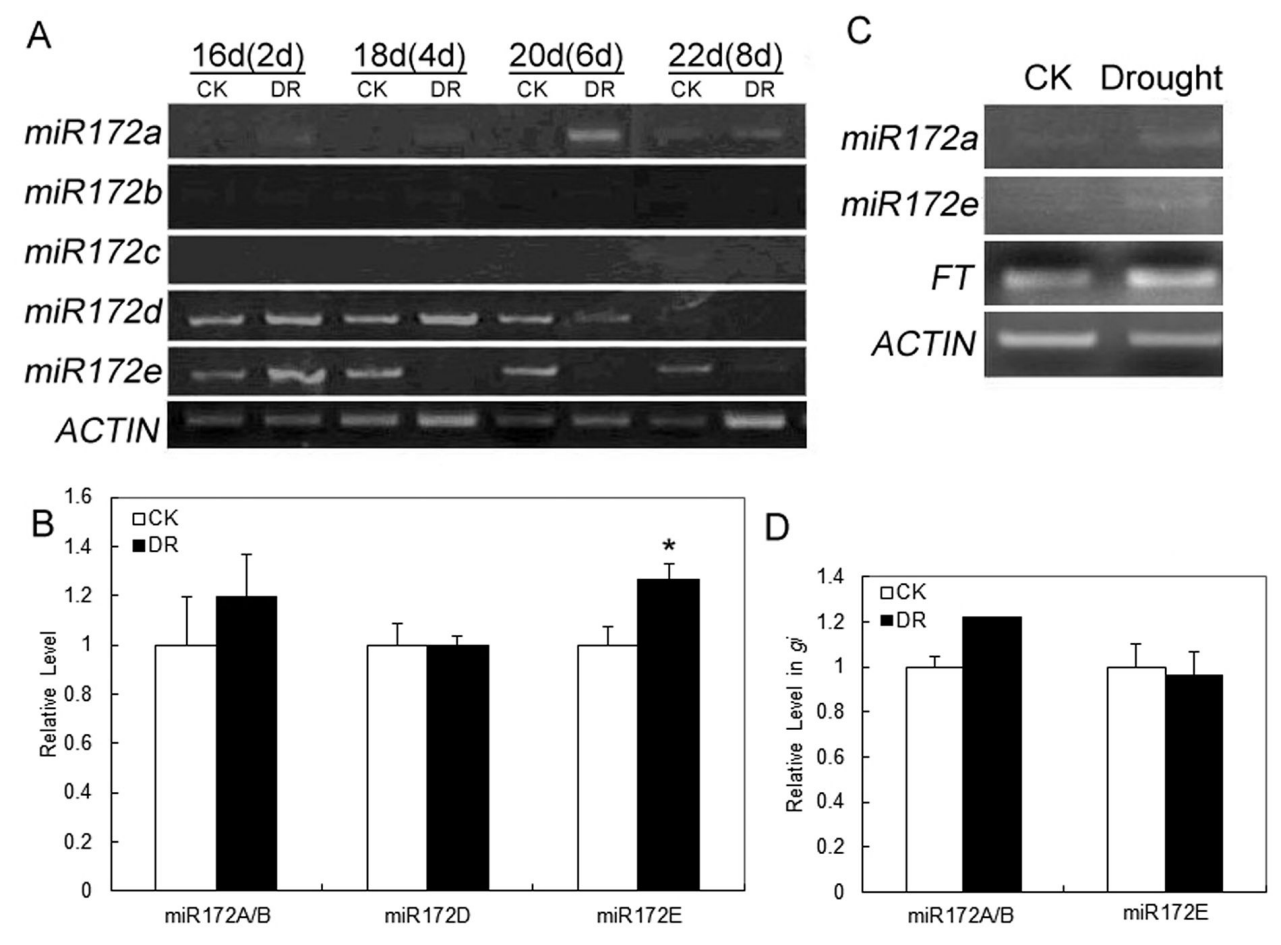
Up-regulation of *miRNA172E* under drought conditions. Each experiment was done triple with similar results. (**A**) Change in *pri-miRNA172* levels under drought conditions( Ler-0). (**B**) Change in mature *miRNA172* levels under drought conditions in wild-type plants. * *P*<0.05. (**C**) RT-PCR analysis of *Pri-miRNA172A* and *Pri-miRNA172E* in the *gi* mutant under drought and control conditions. (**D**) Changes in mature *miRNA172A/B* and *miRNA172E* levels under drought conditions in the *gi* mutant. DR: Drought treatment began from the 14day age. For the mature miRNA assay, samples were collected at the 8^th^ day of DR treatment.

Because the expression of *CO* was not promoted under DR as was that of *GI*, we focused on *miRNA172*, a factor downstream of *GI*. The pri-miRNA level of *miRNA172E* was reduced under DR (Figure 3A), while its mature miRNA level increased (Figure 3B). These data suggested that the processing efficiency of *miRNA172E* was enhanced under drought stress. 

In the *gi* mutant, up-regulation of mature *miRNA172E* (Figure 3D) and down-regulation of *pri*-*miRNA172E* (Figure 3C) under DR were not detected, indicating that the enhanced processing efficiency of *miRNA172E* was dependent on *GI*. 

### *GI* Inhibited the Expression of *WRKY44* under Drought Conditions

**Figure 4:**
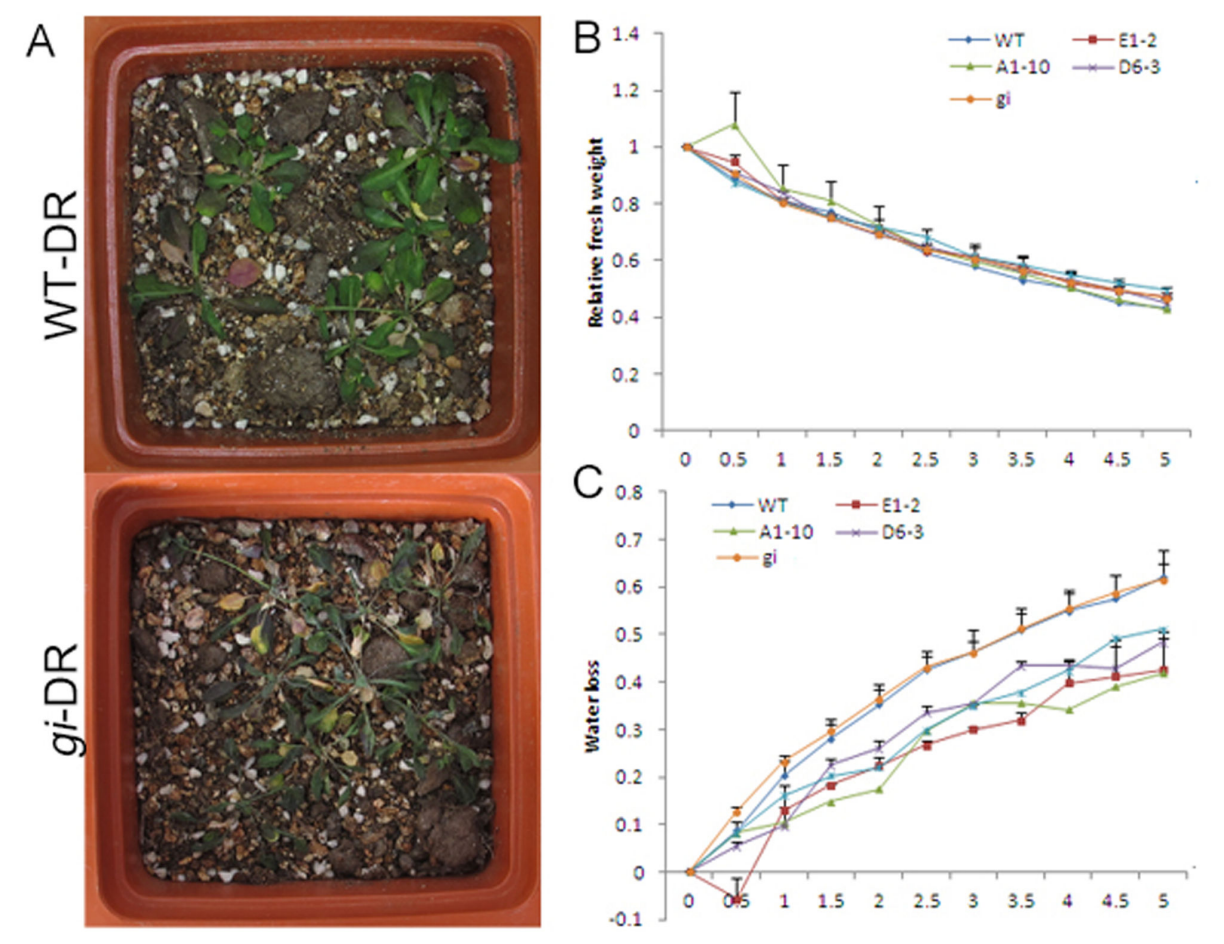
The *gi* mutant is sensitive to drought stress. (**A**) The phenotypes of wild-type plants ( Ler-0) and *gi* mutants under drought stress. (**B**) Transpiration rates of wild type, *gi* and *miRNA172A* (A1-10) */D* (D6-3) */E* (E1-2, E38-6) over-expressing plants. (**C**) Water loss in wild type, *gi* mutants, and plants over-expressing *miRNA172A* (A1-10) */D* (D6-3) */E* (E1-2, E38-6). DR treatment began from10 day age and maintained for 10 days.

In addition to the abnormal drought escape of the *gi* mutant, we observed that *gi* was more sensitive to drought stress than WT (Figure 4A). Although their transpiration rates were similar(Figure 4B), the *gi* plants lost more water than WT in the early stages of dehydration(Figure 4C). The difference was most stark after 0.5 h, while water loss in *gi* and WT was similar at later stages (after 1 h). However, miR172s-OX plants lost much less water than both WT and *gi*. The levels of primary and mature *miRNA172*s in the transgenic plants, as well as their phenotypes, are shown in Figure S1 and flowering times of the transgenic plants were calculated in Table S1. 

Given the higher drought tolerance of *miRNA172* over-expression plants, we can conclude that *GI–miRNA172* may be involved in drought tolerance of *Arabidopsis* (Figure 4A,C). 

**Figure 5:**
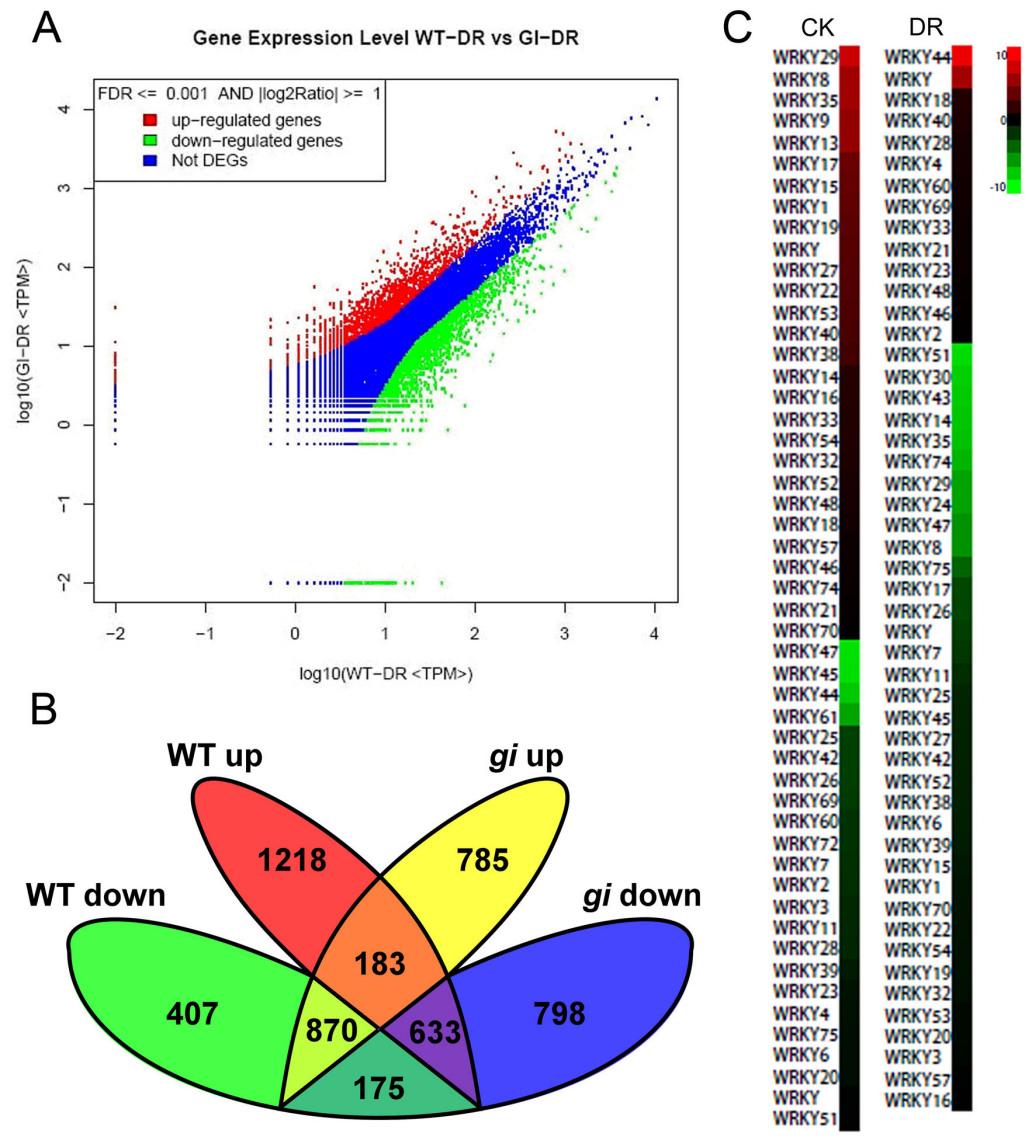
Differential gene expression in wild type (WT) and *gi* mutants under drought conditions as measured by digital gene expression. (**A**) Differential gene expression in WT( Ler-0) and *gi* mutants under drought conditions. (**B**) Venn diagram of up- and downregulated genes in WT and *gi* mutants with and without drought treatment. (**C**) Differential expression of *WRKY* genes in *gi* and WT under CK (standard) and DR(drought) conditions. Red: upregulated in *gi* compared with WT; green: down-regulated in *gi* compared with WT. DR treatment began from10 day age and maintained for 10 days.

DGE analysis was carried out to probe the differentially-transcribed genes in *gi* mutants under DR to gain insight into the relationship between drought defense and escape (Figure 5A). The resulting Venn diagram (Figure 5B) identified cross-talk and differential gene expression between WT and *gi* under CK and DR. Under DR, 1,218 genes were up-regulated in WT but not in *gi*, while 407 were down-regulated in WT but not in *gi*. At the same time, 785 genes were specifically up-regulated and 798 were specifically down-regulated in *gi* under DR. These data implied that some factors were differentially regulated by *GI* under drought stress. 

According to DGE analysis, several *WRKY* (*WRKY DNA-BINDING PROTEIN*) family members exhibited significantly differential expression between *gi* and WT (Figure 5C). Under CK conditions, 20 *WRKY* genes were up-regulated more than two-fold in *gi* compared with WT, while 16 were down-regulated. However, after DR, only four *WRKY* genes had fold increases of two or more in *gi*, while 23 were expressed less (Figure 5C). 

**Figure 6:**
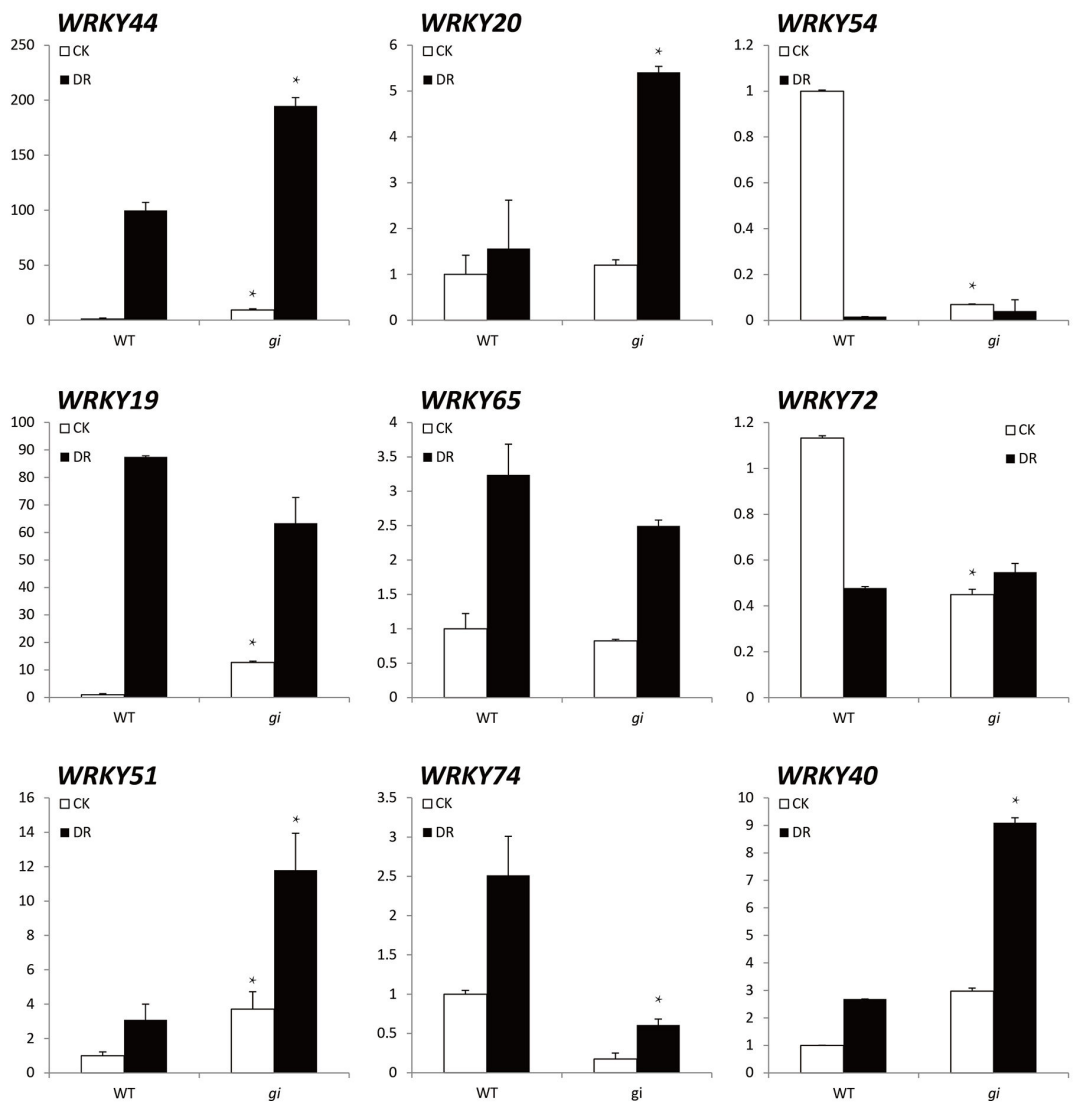
Transcriptional levels of *WRKY* genes in wild type (Ler-0) and *gi* mutants under standard (CK, white rectangles) and drought (DR, black rectangles) conditions. Results are averages of three biological replicates. *, significantly different (*P*<0.05) expression levels between *gi* mutants and wild-type plants under CK or DR. DR treatment began from10 day age and maintained for 10 days.

The expressions of nine *WRKY* family members, representing different subfamilies [34,35], were further examined by qRT-PCR (Figure 6). *WRKY44*, *WRKY 20*, *WRKY 40*, and *WRKY 51* were maintained at much higher levels in *gi* mutants than in WT plants under DR. This finding indicated that *GI* suppressed the expression of these genes under DR. *WRKY44* was unique in that it was also greatly up-regulated (≈ 100-fold) in WT under DR compared to CK, although not as much as in the *gi* mutant (≈ 200-fold). In other words, *WRKY44* was constitutively suppressed by *GI*, while *WRKY20*, *WRKY40*, and *WRKY51* were suppressed only under DR. In contrast, *WRKY54* and *WRKY72* were positively activated by *GI* under CK, while *WRKY74* was positively regulated under both CK and DR. Regulation of *WRKY19* and *WRKY65* seemed to be independent of *GI*.

**Figure 7:**
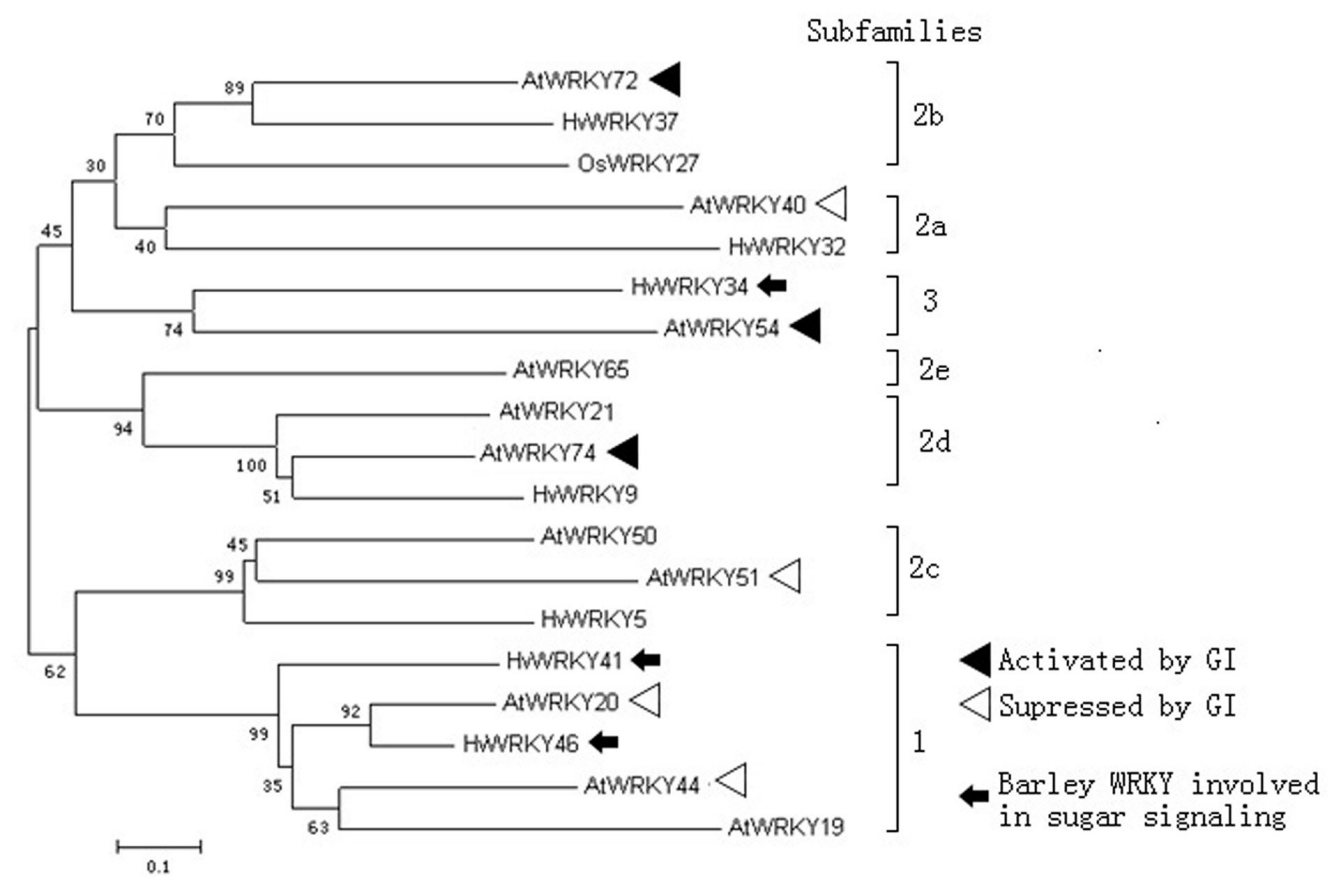
Phylogenetic analysis of *Arabidopsis* *WRKY* genes used in this study and *WRKY* genes from *Hordeum vulgare*. Data were analyzed by the neighbor joining method. Annotations indicate the regulation of *Arabidopsis* *WRKY* genes by *GI*. The number above each branch-point referred to the bootstrap value (maximum is 100), which implied the reliability of existing clades in the tree. The system has performed 1000 replicates to construct the phylogram. The number in each clade represented the percentages of success for constructing the existing clade. 0.1 means 10% substitution rate between two sequences.

Phylogenetically, the *Arabidopsis WRKY*s were classified into two main subfamilies (Figure 7): one included subgroups 1 and 2c and the other the remaining subgroups (2a, 2b, 2d, 2e, 3). According to the qRT-PCR analysis, the genes in subgroups 1 and 2c (e.g., *WRKY20*, *44*, and *51*) were suppressed by *GI* while genes in subgroups 2a, 2b, 2d, 2e, and 3 (e.g., *WRKY54*, *72*, and *74*) were activated by *GI*.

### Interaction between WRKY Protein and TOE1

**Figure 8:**
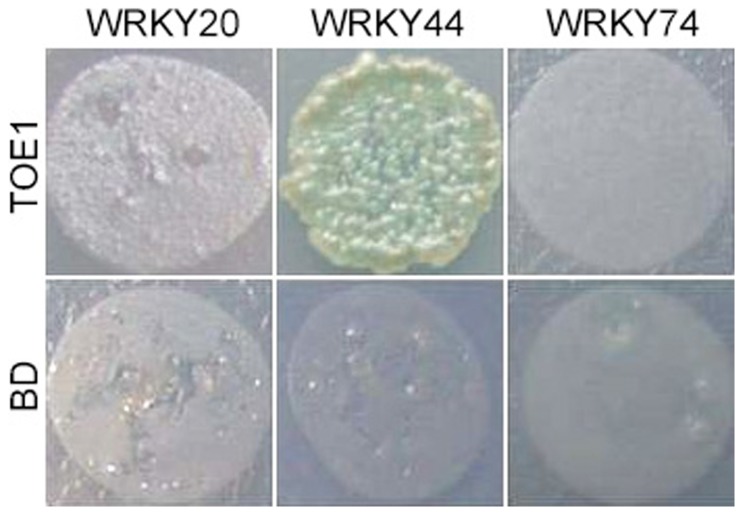
Yeast two-hybrid system analysis of WRKY and TOE1. Using TOE1 as bait identified WRKY44 as a potential protein interactor. Selective plates lacking adenine, histidine, tryptophan, and leucine (–Ade, –His, –Trp, –Leu) and control plates lacking only tryptophan (–Trp) are shown. Empty vectors (BD) and expressed proteins (TOE1) are indicated. Plates were photographed after 4 d. Potential interactors exhibited positive galactosidase activity (blue).

Because a previous report indicated that subgroup 1 was involved in sugar signaling [36], we detected the interaction of members of this subgroup (i.e., *WRKY44* and *WRKY20*) and a member of another subgroup (*WRKY 74*) with *TOE1* (*Target of EARLY ACTIVATION TAGGED* 1), a target of *miRNA172* and suppressor of flowering. In a yeast two-hybrid system, WRKY44, which was suppressed by *GI*, was able to interact with TOE1, while WRKY20 and WRKY74 were not (Figure 8).

**Figure 9:**
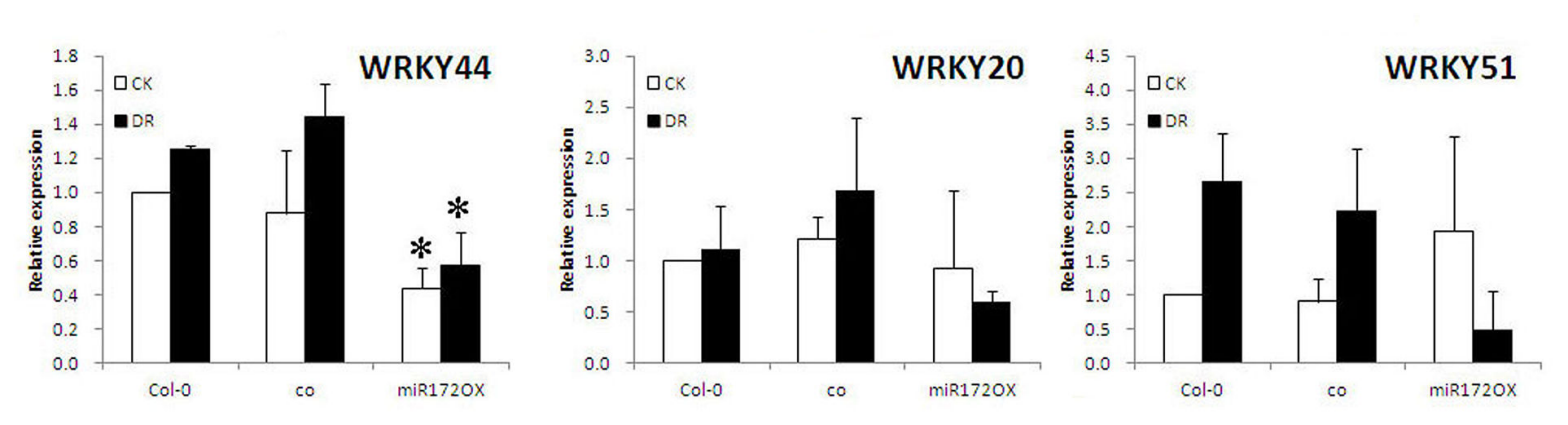
Transcriptional level of *WRKY20*, *WRKY44*, and *WRKY51* in *co* and *miRNA172*–over-expressing plants (miRNA172-OX) under standard (CK, white rectangles) and drought (DR, black rectangles) conditions. Controls for the co mutant and miRNA172-OX was *Col-*0, the wild type in their respective ecotype backgrounds. Results are averages of three biological repeats. * Significantly different (*P*<0.05) expression between miRNA172-OX and WT under both CK and DR conditions. E1-2 line was used as miRNA172-OX. DR treatment began from10 day age and maintained for 10 days.

We examined the expressions of *WRKY44* and its co-members in subgroup 1, including *WRKY20* and *WRKY51*, in *co* mutant and *miRNA172*-OX line(Figure 9). The level of *WRKY44* was significantly reduced in *miRNA172*-OX plants (*P* = 0.015 under CK; *P* = 0.027 under DR) (Figure 9). The levels of *WRKY51* and *WRKY20* were unchanged in *miRNA172*-OX. The down-regulation of *WRKY44* in *miRNA172*-OX plants was consistent with its up-regulation in *gi* mutants, indicating that *GI* and *miRNA172* were in the same pathway suppressing *WRKY44*. But in *co*, the level of WRKYs was similar to WT under both CK and DR conditions (Figure 9). 

## Discussion

Water deficit affects flowering time in many angiosperms [14]. Many plants accelerate flowering under drought conditions, a phenomenon well studied in wheat, *Brassica* and *Arabidopsis* [20,21]. Given that earlier flowering under drought will reduce crop yields, we examined the genetic mechanism of this acceleration in *Arabidopsis*. Our results indicated that the photoperiod factor *GI* might be involved in drought-induced early flowering in *Arabidopsis*. Loss-of-function mutants of *GI* and *CO* could not flower under drought stress. Drought led to increased peak levels of *GI* and *FKF1*. No previous paper has reported a correlation between drought stress and circadian rhythm. But *TOC1* (*Time of CAB expression 1* ), an important gene in circadian control, has been reported to be a molecular switch connecting the circadian clock with plant drought responses via mutual regulation with ABAR (ABA-Related gene) [37]. *GI* and *TOC1* are both circadian regulators, with *GI* activating *TOC1* and *TOC1* repressing *GI* [38,39]. The changed expression of *GI* and its related genes under drought might have resulted from the interaction between *GI* and *TOC1*. 

Drought reduced the peak levels of *FT*, which was unexpected. The down-regulation of *FT* as drought conditions worsened may be related with the increased concentration of endogenous abscisic acid (ABA). We performed an ABA treatment and analyzed the expression of *FT* as described above and found that ABA (50 μM) inhibited the level of *FT* (Figure S2). This finding was consistent with our rhythmical expression data. For example, the reduction of *FT* was not so apparent at the10^th^ d of DR treatment (i.e., the first day of sample collection). But beginning on the 11^th^ day, *FT* levels declined day by day (Figure 2). Nevertheless, by the time samples were collected, the DR-treated plants had flowered, so the reduction of *FT* did not affect the flowering time of *Arabidopsis*. Thus, the suppression of *FT* by DR may result from increased ABA levels in the plant. 

We further investigated *miRNA172*, an important non-coding RNA in the photoperiod pathway that is controlled by *GI* [40]. Although both level and function of *miRNA172* are reported to be enhanced during drought in maize, *Arabidopsis*, and potato (*Solanum tuberosum*) [41-45], differential expression of its family members and other regulating mechanisms have not been studied. In this study, genetic and molecular analyses indicated that *miRNA172*E exhibited the greatest response to drought, with enhanced processing efficiency because of the decreased precursor levels and more mature *miRNA172*E under DR in WT plants. The changes in precursor and mature *miRNA172*E levels in the *gi* mutant indicated that enhanced *miRNA172*E processing under drought was dependent on *GI*. 

The photoperiod pathway senses light via plant aerial parts, especially leaves [41,46,47]. However, water availability is assessed by roots [14]. Water availability signals may be transmitted from roots to leaves. Another group of photoperiod genes, cryptochromes (*CRY*), have been indicated to be related to drought tolerance [48]. In addition, *CRY2* positively regulates *GI* in the photoperiod pathway [49]. The involvement of cryptochromes may explain why *GI*–*miRNA172* was implicated in drought response.

According to our observations, the *gi* mutant was more sensitive and *miRNA172*-OX was less sensitive to drought than WT plants, indicating that *GI–miRNA172* affects drought defenses other than escape. *GI* is known to protect plants from several abiotic stresses, including cold [50] and oxidative stress [50-52]. The involvement of *GI* in drought defense may be related to oxidative stress resulting from dehydration.

Interestingly, some *WRKY* genes, which belong to a defense-related gene family, were characterized as downstream factors of the *GI–miRNA172* pathway. The most significant was *WRKY44*, which was significantly suppressed by *GI* and *miRNA172*. 

Among the targeted genes of *miRNA172*, *TOE1* is the most influential because a single mutant of *toe1* exhibited early flowering [40,53]. We performed yeast two-hybrid screening to detect the interaction of WRKYs and TOE1. WRKY44 could interact with TOE1, a target of *miRNA172*. This further confirmed the regulation of *WRKY44* by *GI–miRNA172*. 

The *WRKY* superfamily can be divided into seven subgroups according to the number of WRKY domains and features of their zinc-finger-like motifs [54]. WRKY44 belonged to subgroup 1 according to our phylogenetic analysis. One recent study indicated that this group of barley (*Hordeum vulgare*) *WRKY*s were involved in sugar signaling [36]. *GI–miRNA172* may be involved in sugar signaling by inhibiting *WRKY44*. Consistent with this hypothesis, excess starch accumulation has been observed in leaves of the *gi* mutant [55], and research indicated that sugar deficiency was responsible for the sensitivity of the *gi* mutant to freezing [56]. *GI–miRNA172* may affect sugar concentration by inhibiting *WRKY44*. 

According to the qRT-PCR, expression of *WRKY*s was not changed in the *co* mutant. Because co exhibited a similar drought phenotype to that of *gi*, and *CO* and *miRNA172* are two independent factors downstream of *GI* [40], *CO* may affect drought escape by regulating other factors. 

**Figure 10:**
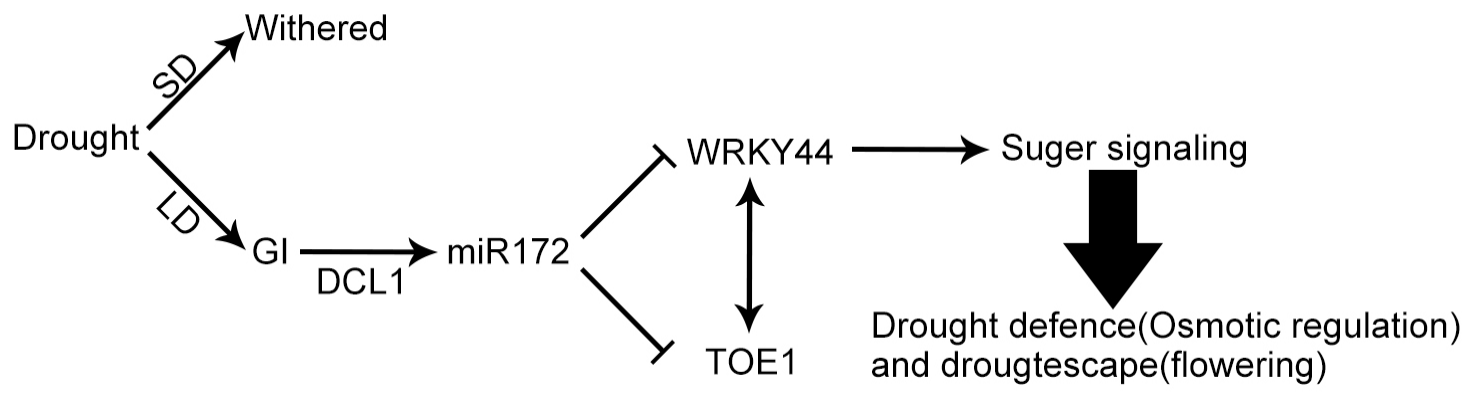
A schematic working model for the involvement of GI and WRKY in drought defense and drought escape in *Arabidopsis*. SD, short day; LD, long day;→, up-regulated; ┥, down-regulated;<->, interact at protein level.

In conclusion, plants prepare to survive increasing drought stress via two strategies. One is to adjust the osmotic potential to defend against impending dehydration. The other is to bloom early to ensure the perpetuation of their genes. Sugar is an ideal signal that can link both strategies, because of its role in both osmotic adjustment [57,58] and the transition from vegetative to reproductive development [59]. This study indicated that *GI*–*miRNA172* and *WRKY* may be factors connecting these two pathways. Figure 10 summarizes a working model of this hypothesis. In the LD and DR condition, increasing peak expression of *GI* promoted the processing of *miRNA172*. *MiRNA172* could suppress the levels of *WRKY44* and *TOE1*, which encode interactive proteins. Because *WRKY44* is involved in sugar metabolism and signaling, *GI–miRNA172* might function in drought escape and defense by affecting sugar signaling. In future studies, the mechanism of interaction between WRKY44 and TOE1 should be examined to investigate the function of TOE1, an important photoperiod factor, in sugar signaling. 

Another important drought-induced factor, ABA, may be involved in both drought responses. ABA can promote drought tolerance in plants [60]. LD conditions promoted ABA levels, indicating that ABA was regulated by the photoperiod pathway [61], but high ABA level will delay flowering [62]. Considering that LD favors flowering, this inconsistency suggests that the concentration of ABA may be a signal for both drought tolerance and drought escape. This is similar to sugar, in that sugar at photosynthetic amounts will promote the floral transition [26], but will inhibit it at excess concentrations [63]. 

## Supporting Information

Figure S1 **The level of pri-*miRNA172*s and mature *miRNA172* in miRNA172-OX plants.** (**A**) The level of pri-*miRNA172a* in miRNA172a-OX plants. (**B**) The level of pri-*miRNA172d* in miRNA172d-OX plants. (**C**) The level of pri-*miRNA172e* in miRNA172e-OX plants. (**D**) The level of mature *miRNA172A* in miRNA172a-OX plants. (**E**) The level of mature *miRNA172D* in miRNA172d-OX plants. (**F**) The level of mature *miRNA172E* in miRNA172e-OX plants. (**G**) The phenotype of miRNA172s-OX plants. A1-10: miRNA172a-OX plants; D6-3: miRNA172d-OX plants; E1-2: miRNA172e-OX plants.
(TIF)

Figure S2 **Suppression of *FT* by ABA treatment.** CK0: Two-week seedling; W12: water-treated seedlings for 12 hr; A12: ABA-treated seedlings for 12 hr; W24: water-treated seedlings for 24 hr; A24: ABA-treated seedlings for 24 hr; and so on.
(TIF)

Table S1 **The flowering time of miRNA172-OX transgenic lines.**
(DOCX)
