# Correction: The Suppression of WRKY44 by GIGANTEA-miR172 Pathway Is Involved in Drought Response of *Arabidopsis thaliana*

**DOI:** 10.1371/journal.pone.0124854

**Published:** 2015-04-06

**Authors:** Yingying Han, Xuan Zhang, Wei Wang, Yaofeng Wang, Feng Ming

Dr. Wei Wang should be listed as third author for this study. Dr. Wang was responsible for analyzing sequences and creating Fig 5B. The authors apologize for this omission. The correct author list is:

Yingying Han, Xuan Zhang, Wei Wang, Yaofeng Wang, Feng Ming

Dr. Wei Wang’s affiliation is:

Department of Biology, Howard Hughes Medical Institute-Gordon and Betty Moore Foundation, Duke University, Durham, North Carolina, United States of America.

The summary of contributions to the study should be revised as below:

Conceived and designed the experiments: FM YH. Performed the experiments: YH XZ. Analyzed the data: YH XZ WW YW FM. Contributed reagents/materials/analysis tools: FM. Wrote the manuscript: YH.      

The correct citation is: Han Y, Zhang X, Wang W, Wang Y, Ming F (2013) The Suppression of WRKY44 by GIGANTEA-miR172 Pathway Is Involved in Drought Response of *Arabidopsis thaliana*. PLoS ONE 8(11): e73541. doi:10.1371/journal.pone.0073541
