# Supplementary figures and images for: The Suppression of WRKY44 by GIGANTEA-miR172 Pathway Is Involved in Drought Response of Arabidopsis thaliana
Source: PLoS One. 2013 Nov 6;8(11):e73541. doi: 10.1371/journal.pone.0073541 (PMC3819348; doi:10.1371/journal.pone.0073541)

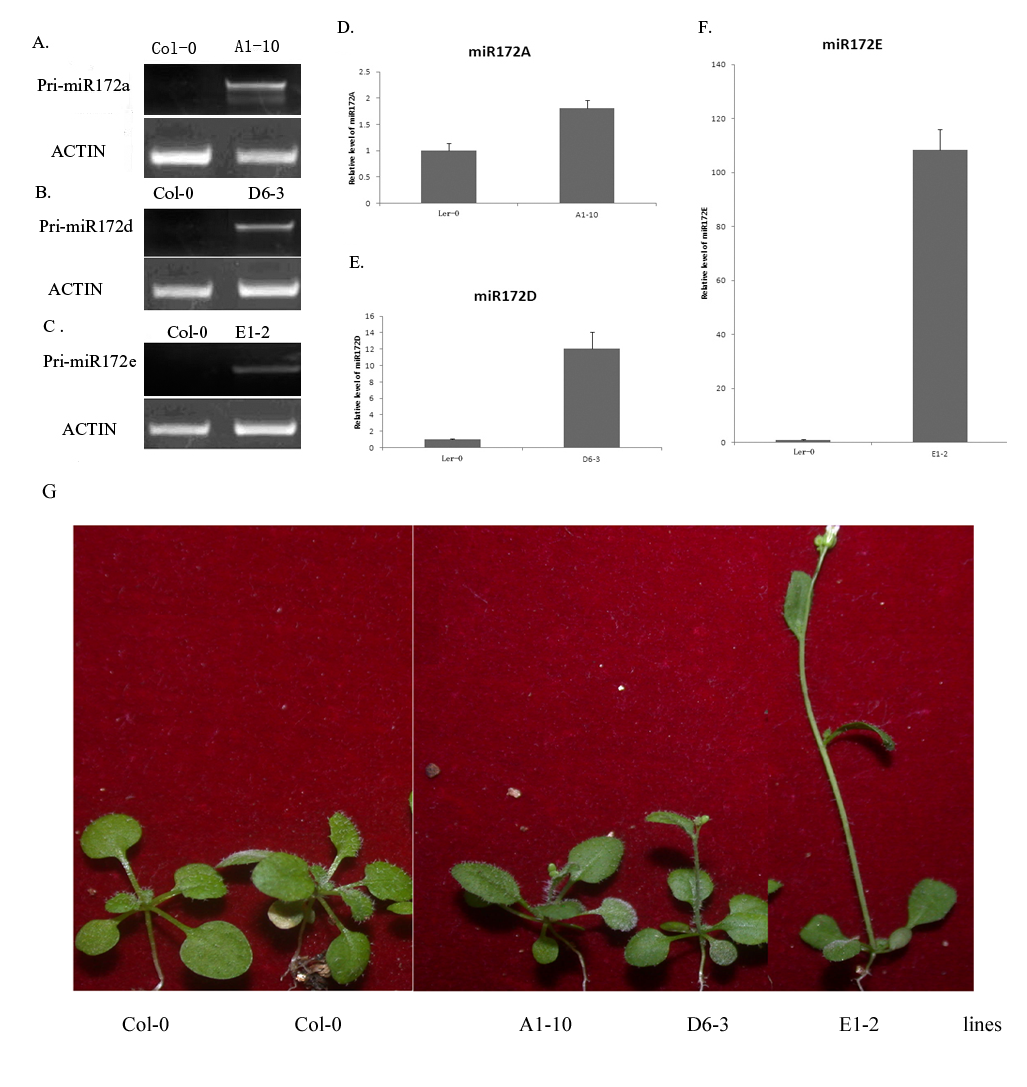

Supplement: Figure S1 — The level of pri-miRNA172s and mature miRNA172 in miRNA172-OX plants. (A) The level of pri-miRNA172a in miRNA172a-OX plants. (B) The level of pri-miRNA172d in miRNA172d-OX plants. (C) The level of pri-miRNA172e in miRNA172e-OX plants. (D) The level of mature miRNA172A in miRNA172a-OX plants. (E) The level of mature miRNA172D in miRNA172d-OX plants. (F) The level of mature miRNA172E in miRNA172e-OX plants. (G) The phenotype of miRNA172s-OX plants. A1-10: miRNA172a-OX plants; D6-3: miRNA172d-OX plants; E1-2: miRNA172e-OX plants. (TIF) [file pone.0073541.s001.tif]

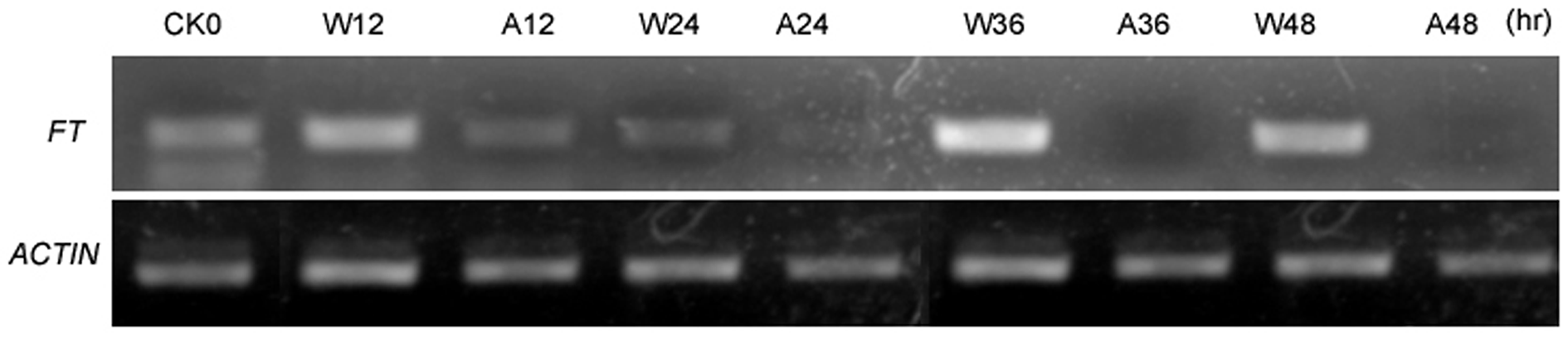

Supplement: Figure S2 — Suppression of FT by ABA treatment. CK0: Two-week seedling; W12: water-treated seedlings for 12 hr; A12: ABA-treated seedlings for 12 hr; W24: water-treated seedlings for 24 hr; A24: ABA-treated seedlings for 24 hr; and so on. (TIF) [file pone.0073541.s002.tif]
