# Supplementary material for: The Suppression of WRKY44 by GIGANTEA-miR172 Pathway Is Involved in Drought Response of Arabidopsis thaliana
Source: PLoS One. 2013 Nov 6;8(11):e73541. doi: 10.1371/journal.pone.0073541 (PMC3819348; doi:10.1371/journal.pone.0073541)
Supplement: Table S1 — The flowering time of miRNA172-OX transgenic lines. (DOCX) [file pone.0073541.s003.docx]

**Supplementary** Table 1．The flowering time of *miRNA172*-OX transgenic lines.

| Transgenic plant | Flowering time under CK | Flowering time under DR |
| --- | --- | --- |
| A1-10 | 16.25 | 13.75 |
| D6-3 | 19.8 | 18 |
| E1-2 | 13.75 | 10.25 |

1. miRNA172A-OX; D. miRNA172D-OX; E. miRNA172E-OX. CK, standard condition; DR, drought condition. The drought treatment began at the 10-d age .
